# Whole-Genome Sequences of SARS-CoV-2 Lineage B.1.525 Strains (Variant η) Detected from Patients in the Abruzzo Region (Central Italy) during Spring 2021

**DOI:** 10.1128/MRA.00618-21

**Published:** 2021-08-05

**Authors:** Emiliano Delli Compagni, Iolanda Mangone, Barbara Bonfini, Annapia Di Gennaro, Liana Teodori, Alessandra Leone, Claudia Casaccia, Ottavio Portanti, Daniela Averaimo, Katiuscia Zilli, Daniela Malatesta, Massimo Ancora, Silvia Scialabba, Marco Di Domenico, Alessio Lorusso

**Affiliations:** a Istituto Zooprofilattico Sperimentale dell’Abruzzo e Molise (IZSAM), Teramo, Italy; DOE Joint Genome Institute

## Abstract

Novel severe acute respiratory syndrome coronavirus 2 (SARS-CoV-2) variants are emerging worldwide. Here, we report the complete genome sequences of 13 severe acute SARS-CoV-2 strains belonging to lineage B.1.525 (variant η).

## ANNOUNCEMENT

On 11 March 2020, the World Health Organization (WHO) declared coronavirus disease 2019 (COVID-19), caused by the zoonotic severe acute respiratory syndrome coronavirus 2 (SARS-CoV-2), a pandemic (1). SARS-CoV-2 belongs to the genus *Betacoronavirus*, the subgenus *Sarbecovirus* included in the subfamily *Orthocoronavirinae*, the family *Coronaviridae*, and the order *Nidovirales*. CoVs are enveloped, single-strand, positive-sense RNA viruses, and they are some of the largest RNA viruses known thus far (2). Several SARS-CoV-2 variants of concern (VOC) and variants of interest (VOI) are circulating globally (3).

Attention should be brought to those mutations occurring in the S protein. This protein permits the binding of SARS-CoV-2 to the ACE-2 receptor and thus is directly involved in the virus-host interaction; it contains a recently described antigenic supersite located in the N-terminal domain (4–6). Therefore, the identification and tracking of mutations through genome sequencing are crucial in order to characterize emerging variants.

Here, we report the complete genome sequences of 13 SARS-CoV-2 B.1.525 (variant η, a VOI) strains identified in the Abruzzo region, central Italy. As part of the legislated mandate of the Italian Ministry of Health (protocol 000644-08/01/2021), the sequencing of these strains did not require approval by any ethics committee. In addition, all tasks were conducted in conformity with the Declaration of Helsinki.

The earliest B.1.525 sequence dates back to 15 December 2020, according to the GISAID global database of coronavirus genome sequences (https://www.gisaid.org), in the United Kingdom. This variant displays a specific set of mutations, with high biological relevance, such as D614G and E484K, which may play a key role in replication, antibody recognition, and vaccine efficacy (7–10).

Nasopharyngeal swab specimens were collected in the Abruzzo region between March and May 2021, and they were sent to the Istituto Zooprofilattico Sperimentale dell’Abruzzo e Molise (IZSAM) for SARS-CoV-2 RNA detection prior to genome sequencing (11). The SARS-CoV-2 diagnosis procedure was performed as previously described (12). An aliquot of 200 μl inactivated nasopharyngeal swab transport medium was employed for RNA extraction. Virus inactivation (PrimeStore molecular transport medium [MTM]; Longhorn Vaccines and Diagnostics, Bethesda, MD, USA) was carried out in a biosafety level 3 biocontainment lab prior to RNA extraction using a MagMAX CORE kit (Thermo Fisher Scientific, Waltham, MA, USA). Detection of viral RNA was performed using the TaqMan 2019-nCoV assay kit v2 (Thermo Fisher Scientific). This assay is designed to target three different regions in the viral genome (ORF1ab, S and N protein-encoding genes). All samples tested positive with threshold cycle (*C_T_*) values of <20 for all targets. Next generation sequencing was carried out using the Illumina COVIDSeq Test (San Diego, CA, USA). Deep sequencing was performed on the NextSeq 500 platform (Illumina, Inc.) using the NextSeq 500/550 high-output reagent cartridge v2, with 75 cycles and 36-bp paired-end format (13).

Quality control of the reads was performed using FastQC v0.11.9 quality control software. The reads obtained were trimmed using Trimmomatic (14). The SARS-CoV-2 reads were mapped to the Wuhan-Hu-1 reference sequence (GenBank accession number NC_045512) using Snippy v4.5.1 (https://github.com/tseemann/snippy). Consensus sequences were obtained using iVar v1.3 (15). All tools were run with default parameters. Details about the obtained reads are listed in Table 1.

**TABLE 1:** Bioinformatics details for each sample

Sample	No. of raw reads	Phred quality score	No. of mapped reads	Coverage depth (×)	Consensus length (bp)	% GC
TE161232/2021	6,666,138	35.44	606,999	7,995	29,688	37.97
TE161257/2021	6,746,522	35.33	775,288	2,978	29,692	37.96
TE161253/2021	6,586,992	35.33	775,236	3,254	29,700	37.97
TE226367/2021	4,213,604	35.44	1,178,896	4,978	29,697	37.98
TE248718/2021	4,307,820	35.44	1,142,716	4,847	29,703	37.98
TE273981/2021	5,458,258	35.33	1,312,159	5,160	29,702	37.98
TE274143/2021	5,117,166	35.33	1,223,245	4,929	29,693	37.98
TE274169/2021	5,575,858	35.44	1,308,171	5,196	29,693	37.98
TE274173/2021	5,643,198	35.44	1,236,729	4,575	29,684	37.98
TE274189/2021	6,554,564	35.44	1,517,988	6,511	29,702	37.98
TE274121/2021	5,213,716	35.33	1,139,139	4,147	29,674	37.98
TE274161/2021	5,199,036	35.33	1,217,514	4,955	29,698	37.98
TE274182/2021	4,975,724	35.33	1,174,421	4,635	29,691	37.98

Multiple sequence alignment of the 13 consensus sequences, using MegAlign PRO (Lasergene; DNASTAR, Madison, WI, USA), showed differences in the nucleotide sequences resulting in missense mutations and therefore in modifications in the amino acid composition. Before submission to the GISAID and NCBI databases, the sequences were uploaded to the Pangolin COVID-19 lineage assigner (https://cov-lineages.org) and assigned to the B.1.525 lineage.

These strains share the same set of mutations with regard to the proteins E, M, N, and NSP6, whereas some mutations are unique to some strains. Interestingly, strain TE274189/2021 is the only one that displays the mutation L513F in the S protein; this mutation is located within the S1 protein but does not belong to the receptor-binding domain (16).

The sequences were released promptly after the sample collection. Here, we want to highlight that the punctual tracking of variants and discovery of novel and relevant mutations are essential strategies for better understanding the virus evolution and transmission and for developing effective countermeasures. Therefore, a global sequencing effort is needed to tackle the COVID-19 pandemic.

### Data availability.

The sequences were deposited in the EpiCoV database in GISAID under the following accession numbers: EPI_ISL_1336084 (hCoV-19/Italy/ABR-IZSGC-161253/2021), EPI_ISL_1336083 (hCoV-19/Italy/ABR-IZSGC-161257/2021), EPI_ISL_1403502 (hCoV-19/Italy/ABR-IZSGC-161232/2021), EPI_ISL_1785083 (hCoV-19/Italy/ABR-IZSGC-226367/2021), EPI_ISL_2001420 (hCoV-19/Italy/ABR-IZSGC-248718/2021), EPI_ISL_2308596 (hCoV-19/Italy/ABR-IZSGC-274182/2021), EPI_ISL_2308595 (hCoV-19/Italy/ABR-IZSGC-274161/2021), EPI_ISL_2308593 (hCoV-19/Italy/ABR-IZSGC-274121/2021), EPI_ISL_2308573 (hCoV-19/Italy/ABR-IZSGC-274189/2021), EPI_ISL_2308572 (hCoV-19/Italy/ABR-IZSGC-274173/2021), EPI_ISL_2308571 (hCoV-19/Italy/ABR-IZSGC-274169/2021), EPI_ISL_2308563 (hCoV-19/Italy/ABR-IZSGC-274143/2021), and EPI_ISL_2308562 (hCoV-19/Italy/ABR-IZSGC-273981/2021). The sequences were also deposited in the NCBI database under the following accession numbers: MZ362439 (TE161257/2021), MZ362440 (TE161253/2021), MZ362441 (TE161232/2021), MZ362442 (TE226367/2021), MZ362443 (TE248718/2021), MZ362444 (TE273981/2021), MZ362445 (TE274121/2021), MZ362446 (TE274143/2021), MZ362447 (TE274161/2021), MZ362448 (TE274169/2021), MZ362449 (TE274173/2021), MZ362450 (TE274182/2021), and MZ362451 (TE274189/2021). The SRA accession numbers are SRX11110421 to SRX11110433.
